# Antibody-dependent CD56+ T cell responses are functionally impaired in long-term HIV-1 infection

**DOI:** 10.1186/s12977-016-0313-6

**Published:** 2016-11-04

**Authors:** Xueying Fan, Liyan Zhu, Hua Liang, Zhe Xie, Xiangbo Huang, Shuo Wang, Tao Shen

**Affiliations:** 1 Department of Microbiology and Center of Infectious Diseases, Peking University Health Science Center, 38 Xueyuan Road, Haidian District, Beijing, 100191 China; 2 State Key Laboratory of Infectious Disease Prevention and Control, National Center for AIDS/STD Control and Prevention, Collaborative Innovation Center for Diagnosis and Treatment of Infectious Diseases, China CDC, Beijing, China

**Keywords:** CD56+ T, ADCC, CD16 cross-linking, Chronic HIV infection, iNKT, MMP inhibitor

## Abstract

**Background:**

Antibody-dependent cellular cytotoxicity (ADCC), which mainly mediated by natural killer (NK) cells, may play a critical role in slowing human immunodeficiency virus type-1 (HIV-1) disease progression and protecting from HIV-1 infection. Besides classic NK cells, CD56+ T cells also have some NK cell-like properties, such as the large granular lymphocyte morphology and the capacity to destroy NK-sensitive target cells. However, little is known about the potentials of antibody-dependent CD56+ T cell responses and the association between antibody-dependent CD56+ T cell responses and HIV-1 disease progression.

**Results:**

In the present study, we showed evidences that, in addition to NK cells, CD56+ T cells could generate degranulation upon CD16 cross-linking. *Ex vivo* study showed that FcγRIII (CD16)-mediated CD56+ T cell responses were distinctly induced by IgG antibody-bound P815 cells. Comparatively, CD56− T cells and invariant NKT (CD3+ 6B11+) failed to induce antibody-dependent activation. Antibody-dependent CD56+ T cell responses were mainly ascribed to CD4/CD8 double negative subset and were functionally impaired in long-term HIV-1-infected former plasma donors, regardless of hepatitis C virus (HCV) coinfection status. Also, CD56+ T cell-mediated HIV-1-specific antibody-dependent responses were declined in men who have sex with men with HIV-1 infection over 3 years. Finally, we showed that matrix metalloprotease (MMP) inhibitor GM6001 could partially restored antibody-dependent CD56+ T cell responses of chronic HIV-1-infected subjects.

**Conclusions:**

Our results suggested that CD56+ T cells could mediate ADCC responses and the responses were impaired in chronic HIV-1 infection.

**Electronic supplementary material:**

The online version of this article (doi:10.1186/s12977-016-0313-6) contains supplementary material, which is available to authorized users.

## Background

Antibody-dependent cellular cytotoxicity (ADCC) is a combined immune responses involving both innate and adaptive immunity [1]. In human, ADCC is typically initiated by recognition of membrane-surface antigens FcγRIIIa (CD16) or FcγRIIc (CD32) of effector cells to the Fc region of IgG antibodies which binding to specific antigens on the surface of target cells [2, 3]. In the last decade, substantial evidences supported the crucial role of ADCC activities in controlling HIV infection. For example, HIV-specific ADCC responses were reported detectable in all tested elite controllers and was significantly higher than in HIV viremic individuals, and ADCC activities were correlated with slowed progression to acquired immune deficiency syndrome (AIDS) in SIV-infected macaques [4–6]. The well-known Thai RV144 HIV vaccine trail showed 31% protection in preventing HIV acquisition with induction of robust HIV-specific ADCC responses [7–9]. In addition to NK cells, other types of circulating granulocytes, such as neutrophils, monocytes and macrophages, were also reported to mediate ADCC activity [10–14].

CD56+ T cells, also termed CD3+ CD56+ NKT-like cells, comprise approximately 5–15% of peripheral circulating T cells in human and express cell-surface molecule-CD56, a typical marker for natural killer (NK) cells [15]. CD56+ T cells are also characterized by some NK cell-like properties, such as the large granular lymphocyte morphology and the capacity to destroy NK-sensitive target cells [15, 16]. Compared with CD56+ T cells, the CD1d-restricted invariant NKT (iNKT) cells, which are characterized by expression of a specific T cell receptor (TCR) (Vα24-Jα18-Vβ11 in humans), also play an important regulatory role in the innate and adaptive immune responses. These iNKT cells share partially phenotypic and functional properties with NK and CD56+ T cells [17]. CD56+ T cells were presumed to display properties of both NK cells and T cells and exhibited capacities of cytotoxicity and cytokine production in both MHC-restricted and MHC-unrestricted manner. CD56+ T cells displayed an essential role in antiviral immune response, such as inhibiting HCV and HIV-1 replication in vitro [18, 19]. However, little is known about the potentials of CD56+ T cells in mediating ADCC responses and the association between CD56+ T cell-mediated ADCC responses and HIV-1 disease progression.

In this study, ex vivo ADCC activities mediated by CD56+ T cells were investigated in healthy versus chronic HIV-1 infected subjects and were compared with NK-mediated ADCC responses. Potenial factors that may influence ADCC activities, such as FcγR expression, CD56+ T subset distribution, and MMPs inhibitor, were also evaluated.

## Results

### CD16 cross-linking triggered degranulation of CD56+ T cells

**Fig. 1:**
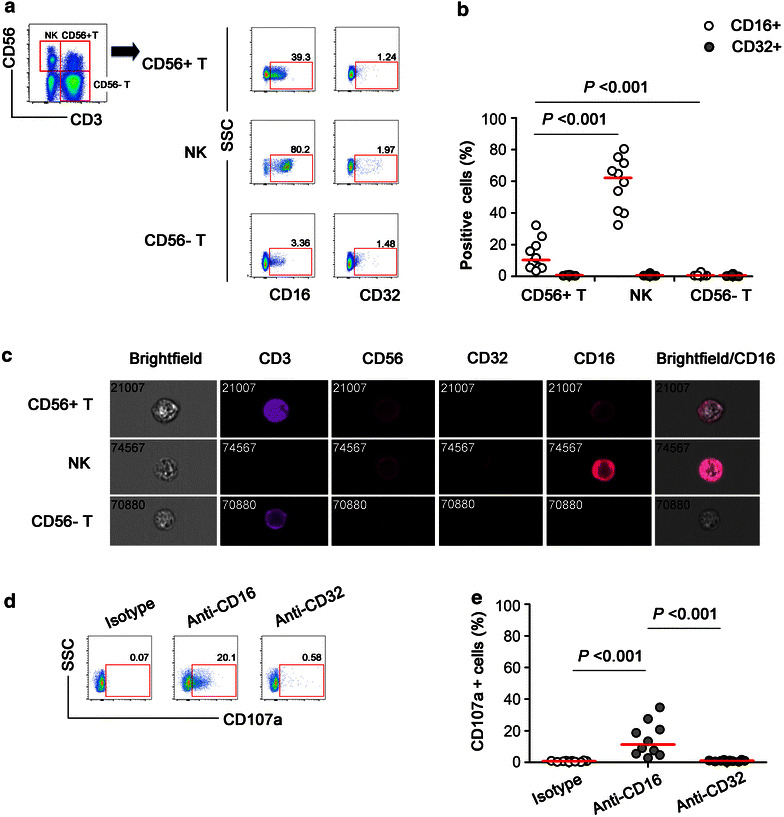
Degranulation of CD56+ T cells could be triggered by CD16 cross-linking. **a** Gating strategies of CD56+ T, CD56+ NK, and CD56− T cells from lymphocytes in a representative subject. The percentages of CD16+ and CD32+ cells in different subsets were also shown. **b** Frequencies of CD16+ and CD32+ cells in CD56+ T, CD56+ NK, and CD56− T cells of healthy donors (n = 10). Data were shown as mean ± SD. **c** Images of CD3, CD56, CD16, and CD32 expression on CD56+ T, CD56+ NK, and CD56− T cells at single-cell level. Data was performed on an ImageStream^χ^ MarkII system. Images of three separate samples were performed with similar results. **d** Representative flow plots depicted the ability of purified CD56+ T cells to degranulate in response to CD16/CD32 cross-linking with anti-CD16, anti-CD32, or nonspecific isotype antibodies. Positive responses were reflected by shifted CD107a expression. **e** The frequencies of CD107a+ CD56+ T cells upon CD16 or CD32 cross-linking in healthy donors (n = 10). The *horizontal bar* indicated median values. All P values were two-tailed and considered significant when less than 0.05

FcγRIIIa (CD16), a low-affinity receptor, could bind to the Fc region of IgG and trigger ADCC responses. Besides CD16, FcγRIIc (CD32) was also reported responsible for ADCC effect [20, 21]. In this study, the frequencies of CD16+ or CD32+ subsets in CD56+ T cells, CD3+ CD56+ NK cells and CD3+ CD56− T cells was identified and compared (Fig. 1a, b). As expected, a considerable percentage of CD56+ T cells was CD16+ CD56+ T cells (11.4 ± 8.1%), which was lower than the percentage on NK cells (60.2 ± 18.9%, *P* < 0.001) but higher than on CD56− T cells (0.9 ± 0.5%, *P* < 0.001). On the contrary, the percentage of CD32+ CD56+ T cells was found very low (less than 2%) on all three cell populations (Fig. 1b). The expression of CD16 and CD32 was confirmed on single cell level by image analysis (Fig. 1c). Purified CD56+ T cells (Purity > 95%, Additional file 1: Fig. S1) were triggered to degranulate after cross-linking with anti-CD16 monoclonal Ab, but not with anti-CD32 Ab (*P* < 0.001, Fig. 1d, e). Taken together, these results indicated that CD56+ T cells expressed a considerable level of CD16 and could be triggered to degranulate after CD16 cross-linking.

### CD56+ T cells could mediate ADCC responses

**Fig. 2:**
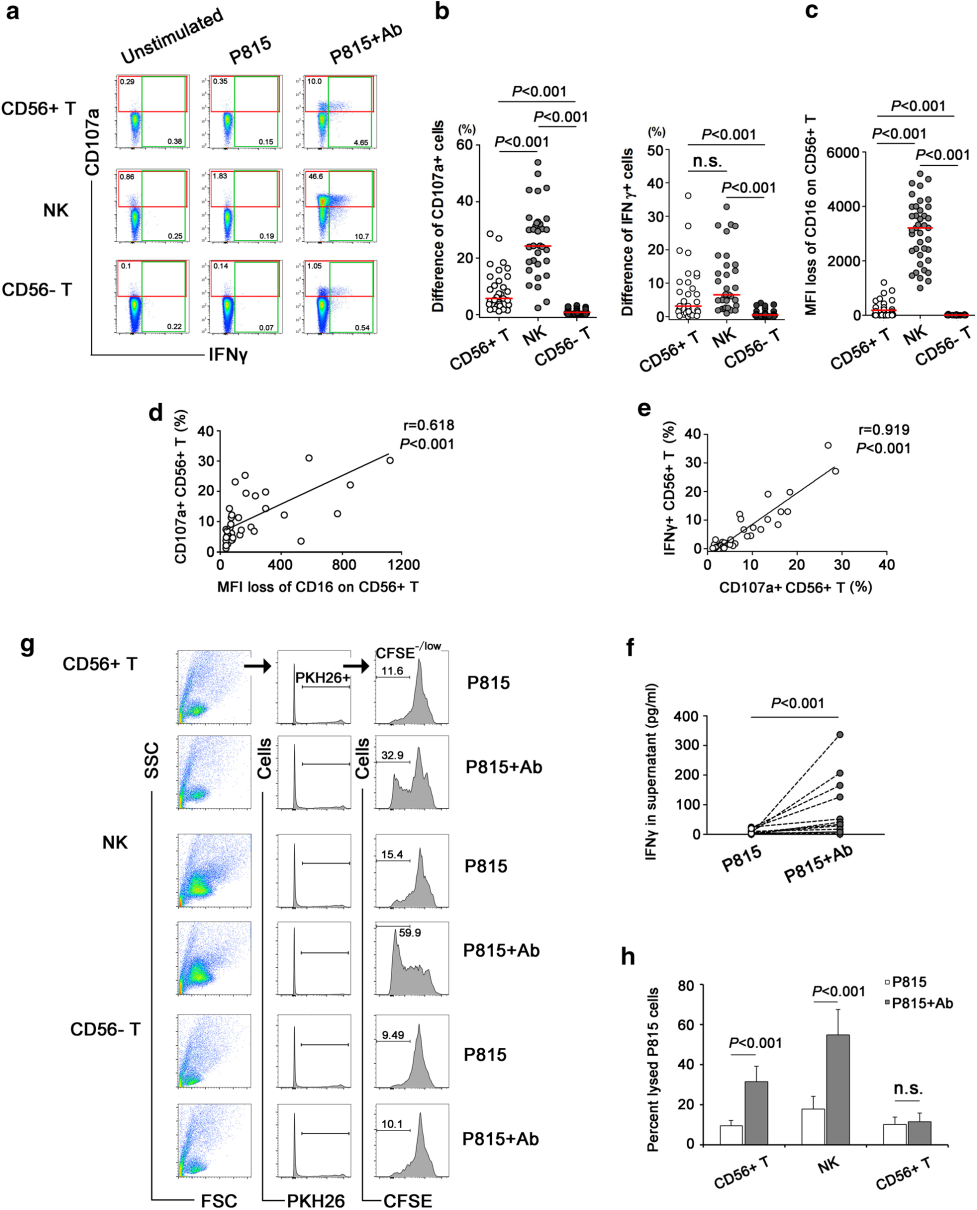
Nonspecific ADCC responses mediated by CD56+ T,CD56+ NK and CD56− T cells. **a** Representative flow plots showing the capacities of CD56+ T,CD56+ NK and CD56− T cells to respond to P815 cells coated with P815-specific Abs (P815+ Ab), p815 cell alone, or medium. Positive ADCC responses were represented by increased CD107a expression and IFNγ secretion. **b** Nonspecific ADCC activities mediated by CD56+ T, CD56+ NK and CD56− T cells were evaluated in 36 healthy donors. The difference of percentage (P815/Ab minus P815) of CD107a+ and IFNγ+ cells in CD56+ T,CD56+ NK and CD56− T cells stimulated by Ab-bound P815 cells was shown. The *horizontal bar* indicated median values. **c** MFI loss (P815 minus P815/Ab) of CD16 on CD56+ T, CD56+ NK and CD56− T cells following stimulation with Ab-bound P815 cells. *Horizontal bar* indicated median value. **d** MFI loss of CD16 was correlated with CD107a+ expression in CD56+ T cell-mediated nonspecific ADCC response. **e** CD107a expression was correlated with IFNγ production in CD56+ T cell-mediated nonspecific ADCC responses. **f** Purified CD56+ T cells were co-cultured with P815 cells or Ab-bound P815 cells for 24 h and levels of IFNγ in cell supernatants were detected by ELISA. **g** Representative flow plots showing lytic capacities of purified CD56+ T, NK, CD56-T cells co-cultured with P815 cells or Ab-bound P815 cells for 10 h. Target P815 cells were shown as PKH26+ cells and CFSE^−/low^ target cells indicated lysed target cells. **h** The percentage of lysed target cells induced by CD56+ T, CD56+ NK and CD56− T cells in healthy donors (n = 10). All P values were two-tailed and considered significant when less than 0.05

In order to extensively detect the capacity of CD56+ cells to mediate ADCC responses, peripheral blood mononuclear cells (PBMCs) were stimulated with P815 cells coated with P815-specific antibodies (P815/Abs) and ADCC response was reflected by CD107a and interferon-γ (IFNγ) production of CD56+ T, CD56+ NK and CD56− T cells. As shown in Fig. 2a, b, IFN-γ and CD107a expression were dramatically elevated in P815/Abs-stimulated CD56+ T and CD56+ NK cells compared with CD56− T cells (*P* < 0.001 for all). In addition, CD56+ T cells expressed a significantly lower CD107a (*P* < 0.001) while similar level of IFNγ when compared with CD56+ NK cells, indicating a weaker degranulation capacity of CD56+ T cells versus NK cells (Fig. 2b). In addition, ADCC response time between CD56+ T and CD56+ NK cells was compared by stimulating effector cells with P815/Abs for 2, 4 and 6 h respectively. As shown in Additional file 2: Fig. S2a and S2b, CD56+ T cells responded to antibody-dependent stimulation after 6 h incubation, while NK cells usually responded after 4 h incubation, which was more quickly than CD56+ T cells.

It is speculated that conjugation of CD16 with Ab-opsonized P815 cells led to down-regulation of CD16 expression and a subsequent cascade of intracellular signal activation, triggering IFNγ and CD107a production. In this study, we found that stimulation with Ab-opsonized P815 cells led to a tremendous decrease of CD16+ population in CD56+ T cells and NK cells, but not in CD56− T subset (Fig. 2c), which was in accordance with their capacities to mediate ADCC response. Further analysis revealed a significant correlation between MFI loss of CD16 and CD107a expression (r = 0.618, *P* < 0.001) in CD56+ T cell-mediated ADCC responses (Fig. 2d). In P815/Abs activated CD56+ T cells, CD107a expression was positively correlated with IFNγ secretion (r = 0.919, *P* < 0.001, Fig. 2e), indicating that cellular degranulation and IFNγ production may share common signal activation pathway in ADCC activities mediated by CD56+ T cells.

To confirm the capacity of CD56+ T cells to mediate ADCC responses, purified CD56+ T cells were co-cultured with P815 cells or Ab-opsonized P815 cells, and supernatants were collected for IFNγ detection by ELISA. As shown in Fig. 2f, the levels of IFNγ were significantly higher in cell supernatant stimulated with Ab-coated p815 cells than with P815 cells alone (*P* < 0.001), confirming that peripheral CD56+ T cells possessed the potential to mediate ADCC function. Moreover, to address antibody-dependent lytic capacity of purified CD56+ T cell, target P815 cells were pre-stained with PKH26 and CFSE, and a rapid fluorometric ADCC (RFADCC) assay was employed to detect the frequencies of CFSE^−/low^ target cells (Fig. 2g). As shown in Fig. 2h, the percentage of lysed P815 target cells were higher in CD56+ T cells incubating with Ab-coated p815 cells than with P815 cells alone (*P* < 0.001). A similar trend of antibody-dependent lytic capacity was found in NK cells but not in CD56− T cells. Taken together, these results indicated that ADCC responses could be mediated by CD56+ T cells though the intensity was generally lower than the response mediated by classic CD56+ NK cells.

### Invariant NKT failed to mediate ADCC responses

**Fig. 3:**
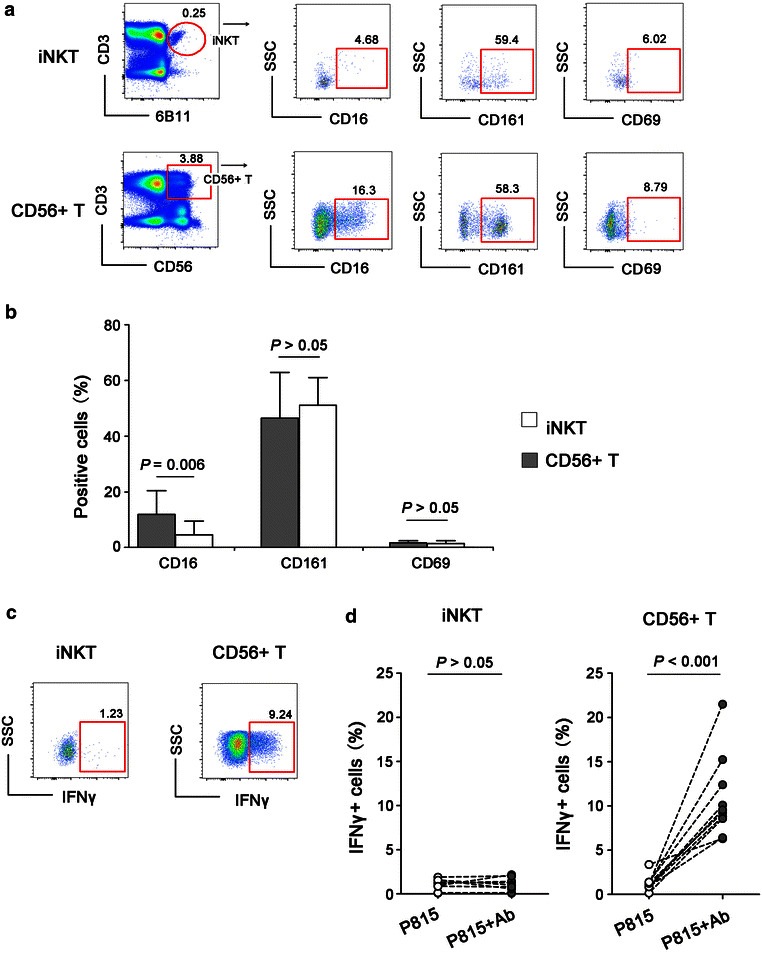
Evaluation of nonspecific ADCC activity mediated by invariant NKT cells. **a** The representative flow plots indicated CD16, CD161 and CD69 expression on human invariant NKT cells (iNKT, CD3+ 6B11+) and CD56+ T cellsrespectively. **b** Comparison of CD16, CD161 and CD69 expression on iNKT and CD56+ T cells from ten healthy donors. **c** The representative flow plots indicated IFNγ production by iNKT and CD56+ T cells stimulated by Ab-bound P815 cells. **d** The frequencies of IFNγ+ iNKT and IFNγ+ CD56+ T cells from ten healthy donors in response to P815 cells alone or Ab-coated P815 cells. All P values were two-tailed and considered significant when less than 0.05

Peripheral iNKT is usually defined as CD3+ Vα24-Jα18+ double positive cells in lymphocytes. Initially, we detected the frequencies of CD16+, CD161+ [22] and CD69+ [23] (two markers of lymphocyte activation) cells in iNKT cells and CD56+ T cells from 10 healthy donors. As indicated in Fig. 3a, b, compared to CD56+ T cells, a significantly lower frequency of CD16+ iNKT cells were found (*P* = 0.006). No differences of CD161+ and CD69+ cells were presented between these two types of cells. Furthermore, the capacity of iNKT to mediate ADCC was evaluated by detecting the frequency of IFNγ+ cells using the strategy for ADCC detection above. Compared to CD56+ T cells, IFNγ-producing iNKT cells were extremely lower after stimulating with Ab-bound P815 cells (Fig. 3c). There was no significant difference in IFNγ production between iNKT cells stimulated with P815 cells alone and Ab-bound P815 cells (*P* > 0.05), in contrast to CD56+ T cells which showed an dramatically increased IFNγ+ production after stimulating with Ab-bound P815 cells (*P* < 0.001) (Fig. 3d). These data demonstrated that, unlike CD56+ T cells, iNKT cells were unable to mediate ADCC responses.

### CD56+ T cell-mediated nonspecific ADCC was impaired in long-term HIV-1 infected FPDs

**Fig. 4:**
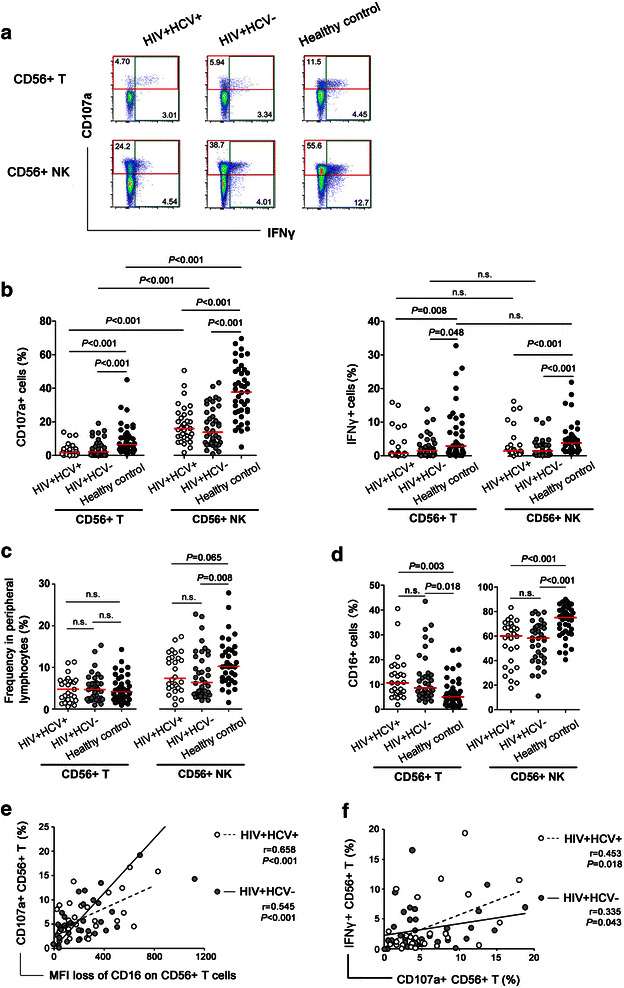
Nonspecific ADCC activities mediated by CD56+ T cells and CD56+ NK cells in long-term HIV-1-monoinfected FPDs, HIV-1/HCV-coinfected FPDs, and healthy donors. **a** Representative flow plots showed the levels of CD107a expression and IFNγ production from CD56+ T cells and CD56+ NK cells in HIV-1 infected subjects and healthy controls. **b** Comparison of percentages of CD107a+ (*left panel*) or IFNγ+ (*right panel*) cells in CD56+ T cells and CD56+ NK cells between HIV-1 infected subjects and healthy controls. **c** The frequencies of CD56+ T cells and CD56+ NK cells in peripheral lymphocytes in HIV-1 infected subjects and healthy controls. **d** The percentages of CD16+ cells in CD56+ T cells and CD56+ NK cells of HIV-1 infected subjects and healthy controls. **e** Correlations between the MFI loss of CD16 and expression of CD107a in CD56+ T cell-mediated nonspecific ADCC in HIV-1/HCV-coinfected (*dash line*) and HIV-1-monoinfected groups (*solid line*). **f** Correlations between CD107a expression and IFNγ production in CD56+ T cell-mediated nonspecific ADCC in HIV-1/HCV-coinfected (*dash line*) and HIV-1-monoinfected groups (*solid line*). The *horizontal bar* indicated median values. All *P* values were two-tailed and considered significant when lower than 0.05

Nonspecific ADCC activity mediated by CD56+ T cells and CD56+ NK cells were evaluated in long-term HIV-1-infected former plasma donors (FPDs) (n = 76, of which 36 were coinfected with HCV) and healthy controls (n = 36). Compared to healthy controls, CD56+ T cell-mediated nonspecific ADCC responses were significantly lower in both HIV-1 monoinfected individuals (*P* < 0.001 for CD107a, and *P* = 0.048 for IFNγ) and HIV-1/HCV coinfected individuals (*P* < 0.001 for CD107a, and P = 0.008 for IFNγ), which was similar to the trends of ADCC mediated by CD56+ NK cells (*P* < 0.001 for all) (Fig. 4a, b). The decreased ADCC activities might be ascribed to the functional impairment of CD56+ T cells, since the frequencies of CD56+ T cells were comparable among HIV-1 monoinfected subjects, HIV-1/HCV coinfected subjects, and healthy subjects (*P* > 0.05, Fig. 4c) and the frequency of CD16+ cells within CD56+ T-cells was even higher in HIV infected subjects than in healthy controls (*P* = 0.018 for HIV-1 monoinfection and *P* = 0.003 for HIV-1/HCV coinfection) (Fig. 4d). No differences were found between HIV-1 monoinfection and HIV-1/HCV coinfection regarding to ADCC activities mediated by either CD56+ T or CD56+ NK cells, suggesting that HCV coinfection had no or negligible influence on nonspecific ADCC activities in HIV-1 infection.

In addition, the positive correlations between frequencies of CD107a+ CD56+ T cells and MFI loss of CD16 on CD56+ T cells still existed in both HIV-1-monoinfected (r = 0.545, *P* = 0.007) and HIV-1/HCV-coinfected (r = 0.658, *P* < 0.001) subjects (Fig. 4e). Similarly, positive correlations between frequencies of CD107a+ CD56+ T cells and IFNγ+ CD56+ T cells were presented in HIV-1 monoinfection (r = 0.335, *P* = 0.043) and HIV-1/HCV coinfection (r = 0.453, *P* = 0.018) (Fig. 4f). These results indicated that CD56+ T cells sustained the balance of cytotoxicty and immune regulation, though with an impaired capacities to mediated immune responses. No significant correlation between CD56+ T cell-mediated ADCC activities and HIV-1 viral loads or CD4+ T cell counts were found (data not shown).

Taken together, these data suggested that HIV-1 infection impaired the capacity of nonspecific CD56+ T cells to mediate ADCC responses in long-term HIV-1+ subjects, while HCV coinfection did not aggravate the impairment.

### HIV-1-specific ADCC activities mediated by CD56+ T cells were declined in MSM with HIV-1 infection over 3 years

**Fig. 5:**
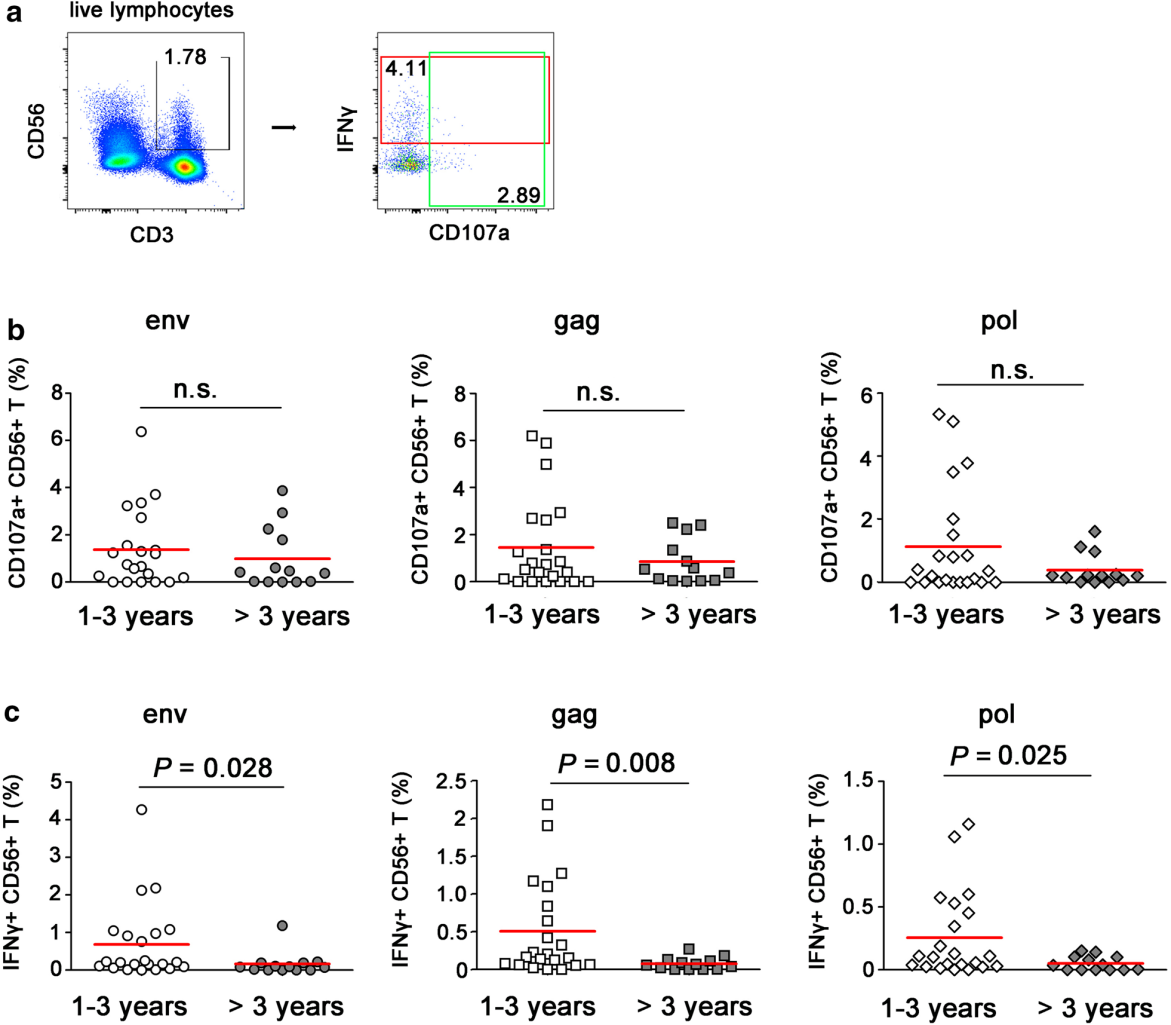
Comparisons of HIV-1-specific CD56+ T-mediated ADCC activity in men who have sex with men (MSM) with HIV-1 infection between 1 and 3 years (n = 22) and >3 years (n = 13). **a** The representative flow plots indicated CD107a expression and IFNγ production by CD56+ T cells. HIV-1-specific ADCC responses were compared between MSM with HIV-1 infection for 1–3 years and >3 years. Whole blood samples were incubated at 37 °C for 5 h with HIV-1 gag, pol, and env peptide pools respectively. HIV-1-specific ADCC responses were identified by expression of CD107a (**b**) and IFNγ (**c**) in CD56+ T cells. The *horizontal bar* indicated median values. All *P* values were two-tailed and considered significant when lower than 0.05

HIV-1-specific ADCC activities were evaluated in men who have sex with men (MSM) subjects with HIV-1-infection for 1–3 years (n = 22) and over 3 years (n = 13). HIV-specific ADCC responses were identified by the frequencies of CD107a+ or IFNγ+ CD56+ T cells in response to HIV-1 specific peptide pools (Fig. 5a). In MSM with HIV-1-infection over 3 years, intracellular IFNγ expression by CD56+ T cells was dramatically declined in comparison with in subjects infected for 1-3 year (env: *P* = 0.028, gag: *P* = 0.008, and pol: *P* = 0.025, Fig. 5c). Though similar trends were found in the frequency of CD107a+ CD56+ T cells in response to individual peptide pool, no significant *P* values were reached (Fig. 5b). These data indicated that CD56+ T cell-mediated ADCC was impaired in chronic HIV-1-infected subjects but not recently infected subjects, which was consistent with the impaired capacities of CD56+ T cells to mediate nonspecific ADCC responses in long-time HIV-1 infected FPDs.

### CD56+ T cells mediating ADCC activities were mainly CD4/CD8 double negative subset

**Fig. 6:**
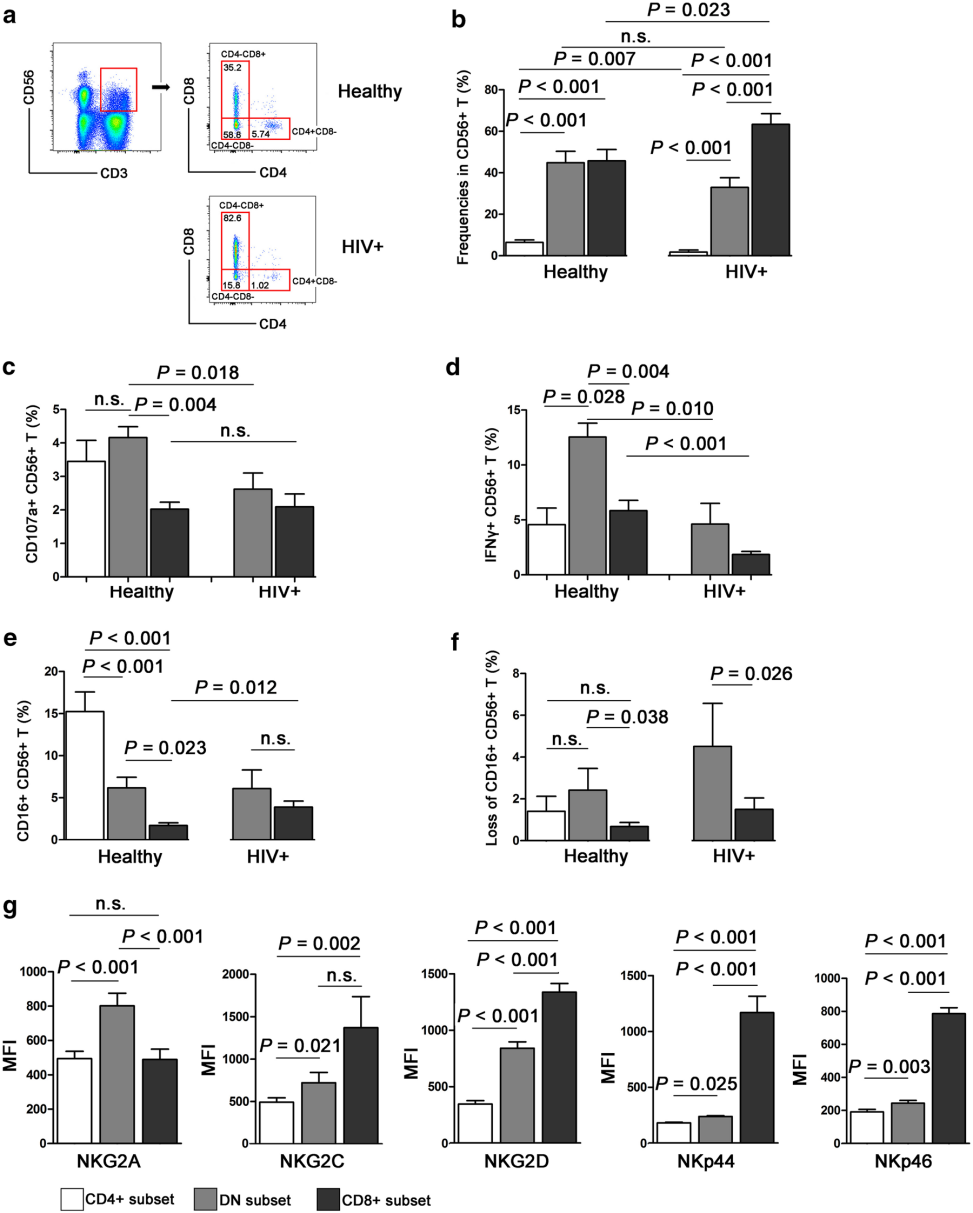
ADCC activity mediated by CD56+ T cells were mainly ascribed to CD4/CD8 double negative subset. **a** CD56+ T cells were divided into three subsets: CD4+ CD8−, CD4− CD8+, and CD4− CD8− (double negative, DN) subsets. Representative flow plots from a HIV-infected individual and a healthy donor were shown. **b** Frequencies of different CD56+ T subsets in HIV-1+ (n = 10) and healthy (n = 10) subjects. **c**, **d** ADCC activities mediated by CD56+ T subset from HIV-1+ and healthy subjects were evaluated and compared. CD107a expression (**c**) and IFNγ production (**d**) of CD4+ CD8−, CD4− CD8+, and DN subset activated by Ab-bound P815 cells in HIV-1+ (n = 10) and healthy (n = 10) subjects were compared. **e** Comparison of CD16+ cells in individual CD56+ T subsets in HIV-1+ and healthy subjects. **f** Loss of CD16 MFI on different CD56+ T subsets in HIV-1+ and healthy subjects. **g** Expressions of NKG2A, NKG2C, NKG2D, NKp44 and NKp46 on three CD56+ T subsets from ten healthy subjects. *MFI* mean fluorescence intensity. **b**–**g** Data were showing as mean ± SD

According to the surface expression of CD4 and CD8, CD56+ T cells could be divided into three subsets: CD4+ CD8− (CD4+ subset), CD4− CD8+ (CD8+ subset), CD4− CD8− (double negative subset, DN) (Fig. 6a). In healthy subjects, the proportion of DN subset and CD8+ subset was similar, occupying appropriate 40% of total CD56+ T cells respectively, while the frequency of CD4+ subset was significantly lower than DN and CD8+ subsets (*P* < 0.001, Fig. 6b). In long-term HIV-infected subjects, the frequency of CD4+ subset was significantly decreased (*P* = 0.007), while the frequency of CD8+ subset was increased (*P* = 0.023) when compared to healthy controls (Fig. 6b). No difference in percentage of DN subset was found between HIV-infected FPDs and healthy controls (*P* > 0.05, Fig. 6b).

Next, ADCC activities mediated by different CD56+ T subsets were evaluated. As shown in Fig. 6c, d, DN subset contributed to the highest CD107a expression (DN vs. CD8+: *P* = 0.004) and IFNγ production (DN vs. CD4+: *P* = 0.028; DN vs. CD8+: *P* = 0.004) in healthy controls, though CD16+ frequency was lower on DN subset compared with CD4+ subset (*P* < 0.001, Fig. 6e). In long-term HIV-1 infection, ADCC activities mediated by DN CD56+ T subset were significantly declined (CD107a: *P* = 0.018; IFNγ: *P* = 0.010), indicating a functional impairment of the subset (Fig. 6c, d). The favorable ADCC activities mediated by DN CD56+ T subset was confirmed by the most loss of CD16 (frequencies) on DN subset than CD8+ subsets in both HIV-1-infected subjects (*P* = 0.026) and healthy controls (*P* = 0.038) (Fig. 6f). Of note, for HIV-1 infection, the characteristics of CD4+ subset and its capacity to mediate ADCC responses were missed in the study due to a very low cells number resulting unreliable results. Taken together, these results suggested that ADCC response mediated by CD56+ T cells was mainly ascribed to DN subset and was significantly impaired in long-term HIV-1 infection.

Finally, in consideration of different performances of three subsets of CD56+ T in mediating ADCC activities, we evaluated the characteristics of CD56+ T subsets in expressing NK cell-associated markers, including NKG2A, NKG2C, NKG2D, NKp44 and NKp46. The data showed a good consistency that the highest levels of activate receptor NKG2C, NKG2D, NKp44 and NKp46 were observed on CD8+ subset, and moderate on DN cells and the lowest on CD4+ subset (Fig. 6g). However, inhibitory receptor NKG2A was found highest on DN subset (*P* < 0.001) (Fig. 6g).

### MMP inhibitor could partially restore CD56+ T cells mediated ADCC in long-term HIV-1 infected subjects

**Fig. 7:**
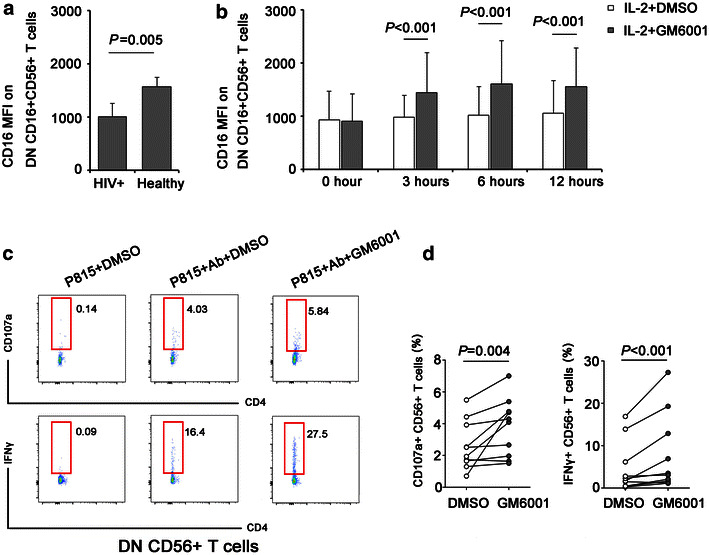
MMP inhibitor GM6001 partially restored the impaired ADCC capacity mediated by DN CD56+ T subset of HIV-1+ subjects. **a** CD16 MFI on DN CD16+ CD56+ T cells were compared between HIV+ patients (n = 10) and healthy controls (n = 10). **b** CD16 MFI on DN CD56+ T cells activated by IL-2+ DMSO and IL-2+ GM6001 for 0, 3, 6, 12 h. **c** CD107 expression and IFNγ production of DN subset from HIV+ patients (n = 10) activated by P815+ DMSO, P815+ Ab+ DMSO and P815+ Ab+ GM6001 were determined by flow cytometry. **d** The percentage of CD107a+ CD56+ T cells and IFNγ+ CD56+ T cells activated by Ab-bound P815 cells in the presence or absence of GM6001 from HIV-1+ subjects (n = 10)

Activation of NK cells induces matrix metalloproteinase (MMP)-mediated cleavage of cell surface CD16 [24–26]. MMP inhibitor was reported to improve the ability of NK cells to mediate ADCC [27]. In this study, we found that CD16 MFI on DN CD16+ CD56+ T cells was lower in HIV+ patients versus healthy donors (*P* = 0.005) (Fig. 7a) and could be reversed by GM6001 treatment for 3, 6 and 12 h (*P* < 0.001, Fig. 7b). To evaluate whether MMP inhibitor could improve CD56+ T cells mediated ADCC in HIV-1 infection, purified DN CD56+ T cells from 10 long-term HIV-1 infected patients were cultured with Ab-coated P815 cells in the presence or absence of broad-spectrum MMP inhibitor GM6001. The results indicated that MMP blockade increased the intensity of degranulation as measured by percentage of CD107a+ cells (*P* = 0.004) and enhanced the secretion of IFNγ (*P* < 0.001) (Fig. 7c, d). Therefore, MMP inhibitor treatment partially restored the impaired ADCC responses mediated by CD56+ T cells during long-term HIV-1 infection. In addition, we found GM6001 failed to enhance antibody-dependent CD56+ T response in healthy donors (data not shown), indicating that MMP inhibitor increases the potentials of CD56+ T cells to mediate antibody dependent responses only in a disease-specific manner.

## Discussion

Accumulated evidence showed that antibody-dependent cellular cytotoxicity may play an important role in the control of HIV-1 infection [28–32]. However, whether CD56+ T cells could act as effector cells to mediate ADCC response is not clearly determined. CD56+ T cells were reported to produce large quantities of IFNγ and played an important role in natural outcome after exposure to HIV-1 or HCV [33–35]. For example, Montoya et al. reported that the frequencies of IFNγ-producing CD56+ T cells activated by PMA/ionomycin were significantly higher in HIV-exposed seronegative (HESN) individuals than in chronic HIV-1-infected subjects [33]. In addition, IFNγ production stimulated by PMA/ionomycin was much higher in CD56+ T cells than in NK cells and common CD3+ T cells [33]. Kokordelis et al. demonstrated that activated CD56+ T cells could inhibit HCV replication in vitro in an IFNγ-dependent manner [19]. Exposure to HCV has been associated with high level of IFNγ production and degranulation of CD56+ T cells [35, 36]. In the present study, we showed that CD56+ T cells have the capacity to degranulate after cross-linking with anti-CD16 antibody and could mediate ADCC responses in vitro. The characteristics of CD56+ T cell-mediated ADCC responses were somewhat different from ADCC activities mediated by classic CD56+ NK cells, since NK-mediated ADCC was marked by both powerful degranulation and IFNγ production while degranulation capacity was significantly weaker in CD56+ T cell-mediated ADCC though a comparable level of IFNγ production was observed.

iNKT cells was identified as CD3+ Vα24-Jα18(6B11)+ cells and responded to CD1d-restricted lipid ligands [37]. Different from CD56+ T cells, we found iNKT expressed less CD16 than CD56+ T cells and failed to response to Ab-coated P815 cells, indicating that iNKT was unable to mediate ADCC responses though shared some NK characteristics. Another study has also found that iNKT cells did not mediated killing of neuroblastoma cell lines in the presence of a relevant antibody, although ADCC of mediated by NK cells was enhanced by activated iNKT cells [38]. CD56+ T cells were heterozygous cell population and could be subdivided into CD4+, CD8+, and DN subsets. Interestingly, unlike CD56− T cells, in which CD4+ or CD8+ single positive subset played the dominant roles in mediating immune response, DN subset within CD56+ T cells occupied a dominant frequency of total CD56+ T cells and mediate the most robust ADCC activities. We speculated that DN subset was inclined to a NK-like function due to its negative expression of CD4 and CD8 and was unable to activate in a MHC-dependent manner. In addition, nature killer cell receptor (NKR) patterns were quite different among CD4+, CD8+, and DN subsets. CD4+ subset is characterized by the weakest expressions of NK cell-distinctive markers (NKG2A, NKG2C, NKG2D, NKp44 and Nkp46), which are expressed highest on CD8+ cells subset (except NKG2A), and then DN cells. The moderate frequencies of both CD16 and NK cell-specific markers may contribute to the highest ADCC activity mediated by DN subset.

In this study, we found the capacity of CD56+ T cells to mediate nonspecific ADCC function in long-term HIV-infected FPDs was significantly weaker than in healthy controls. The decreased CD56+ T cell-mediated ADCC activities in HIV-1 infection might be ascribed to the functional impairment since the frequencies of CD56+ T cells were comparable among groups (Fig. 4c). In addition, frequencies of CD16+ CD56+ T cells were found higher in HIV-1 infected subjects, which was different from the reduced frequency of CD16+ NK cells in HIV-1+ patients as reported previously and observed in this study [27, 39]. Considering that frequencies of CD16+ CD56+ T cells in DN subset was similar between HIV-1 infected subjects and healthy control, increased frequencies of CD16+ CD56+ subset in CD8+ CD56+ T cells was the main source of elevated CD16 on bulk CD56+ T cells in HIV-1+ subjects. Due to the loss of CD4+ T cells in HIV-1 infection, we also found a decreased frequency of CD4+ CD56+ T cells in HIV-1 infection. As a result, the frequency of CD8+ CD56+ T cells was increased. However, CD8+ CD56+ T subset showed less capacity to mediate ADCC compared with DN subset. Combined the characteristics of less frequency, higher CD16 expression, moderated activate NKR expression, and highest ADCC activity of DN subset, we speculated that a balance between expression of surface CD16 and NKR might determine the ADCC capacities of different CD56+ T subsets.

Theoretically, nonspecific ADCC activities detected by Ab-opsonized P815 cells merely evaluated the potentials of effector cells to mediate degranulation and cytokine secretion, while failed to consider other important participants involved in ADCC response, such as Ab factor. In this study, we employed a HIV-1-specific CD56+ T cell-mediated ADCC assay using whole blood samples incubating with HIV-1 peptide pools to identify HIV-1-specific ADCC activities in HIV-1-infected individuals. Our data indicated that HIV-1-specific ADCC activity was also declined in MSM with HIV-1 infection longer than 3 years. To rule out TCR-dependent CD56+ T responses, whole blood samples used in HIV-1-specific ADCC assay were replaced by PBMCs samples. The results indicated that TCR-dependent CD56+ T cell responses (indicated by intracellular IFNγ production) were significantly lower than TCR-dependent CD56− T cell responses (Additional file 3: Fig. S3). The mean values of IFNγ producing CD56+ T cells stimulated by env, gag and env were all less than 0.2%, which were much lower than HIV antibody-dependent CD56+ T responses and could be ignored in the CD56+ T-mediated HIV-1-specific ADCC study. Moreover, the impaired ADCC activities mediated by CD56+ T cells may not only ascribe to the changes of phenotype, functionality and subset distribution of CD56+ T cells per se, but also associate with the alteration of titer, subclassand clonal natureof antibody involved in ADCC responses [5, 40–43].

## Conclusions

In summary, our finding indicated that CD56+ T cells from long-term HIV-1-infected patients displayed weaker antibody-dependent capabilities of cytotoxicity and IFNγ production. The impaired ADCC activities mediated by CD56+ T cells might represent a novel mechanism of dysregulated immune response in chronic HIV-1-infected patients, which shed light on the development of new target for HIV vaccine design or immune therapy.

## Methods

### Study population

A total of 111 HIV-1-infected participants and 36 healthy controls were recruited in this study. Fasted venous blood samples were collected from each participant. Routine blood tests, clinical biomedical tests, and CD4 +/CD8+ T cell counts were performed by local CDC. Peripheral blood mononuclear cells (PBMCs) were prepared from fresh EDTA anti-coagulated peripheral blood using Histopaque-1077 (Sigma, 10,771–500 ml) as described elsewhere [21] and were stored (5 × 10^6^ cells per vial) in liquid nitrogen till use. Serum and EDTA anti-coagulated plasma were stored at −80 °C until use.

HIV-1 infection was screened by an ELISA assay (GBI Biotech, Beijing, China) for HIV antibody, and positive tests were confirmed by HIV Blot 2.2 WB assay (Genelabs Diagnostics, Singapore). The status of anti-HCV antibodies was determined using the ARCHITECT Anti-HCV System (Abbott Diagnostics, Abbott Park, USA). Plasma level of HIV-1 RNA was measured with the Standard Amplicor HIV Monitor assay, version 2.0 (Roche Diagnostics, Indianapolis, IN, USA), according to the manufacturer’s protocols. The lower limit of detection was 40 copies/ml. Plasma HCV RNA level was quantified with 2nd Cobas Ampliprep/CobasTaqman (CAP/CTM) HCV test system (Roche, Branchburg, NJ, USA) with a detection limit of 15 IU/ml.

#### FPDs and healthy controls

The study involved 76 anti-HIV-positive former plasma donors (FPDs) (Table 1), recruited from a village in central China in July 2012. Among the 76 HIV-1+ individuals, 36 were coinfected with HCV and 40 were infected by HIV-1 alone. The infection time of these HIV-1+ patients was highly uniform (13–16 years), and all individuals were infected by a single closely related HIV-1 strain via unregulated commercial plasma donation practices in the late 1990s. HCV infection was characterized by positive anti-HCV response and positive results for HCV-RNA detection. All recruited subjects were negative for hepatitis B surface antigen (HBsAg) and had never received any forms of HCV-specific antiviral therapy. All HIV-1-positive FPDs had received regular or intermittent first-line antiretroviral therapy. 36 healthy adults from the same village who were negative for HBV/HCV/HIV-1 infection were recruited as controls (Table 1).

**Table 1:** Characteristics of HIV-1-infected former plasma donors (FPDs) and control individuals

Characteristics	HIV-1-monoinfected	HIV-1/HCV-coinfected	Healthy
Number	40	36	36
Female*	25 (62.5)	20 (55.6)	18 (50.0)
Age (years)^†^	43 (9.4)	44 (9.3)	46 (10.2)
Deduced infection time (years)^†^	15.1 (1.5)	14.8 (1.5)	NA^a^
Clinical data			
ALT (IU/L)^†b^	22.5 (25.1)	33.0 (50.5)	19 (12.0)
AST (IU/L)^†c^	29.0 (24.9)	35.5 (57.5)	22 (11.6)
HIV-status			
Anti-HIV	Positive	Positive	NA
HIV RNA (copies/ml)^†^	11,452 (8933)	11,075 (7866)	NA
CD4 cells/μl^†^	384 (234)	403 (233)	NA
CD8 cells/μl^†^	936 (394)	850 (517)	NA
HCV-status			
Anti-HCV	Negative	Positive	NA
HCV RNA (log_10_ IU/ml)^†^	0 (0)	6.4 (0.9)	NA
HCV genotype			
1b*	NA	15 (41.7)	NA
2a*	NA	20 (55.6)	NA
Others*	NA	1 (2.7)	NA

* Number of cases (number/total in %)

^†^ Mean ± standard deviation (SD)

^a^Not applicable, ^b^ alanine aminotransferase, ^c^ aspartate aminotransferase

#### MSM

35 HIV-1 positive men who have sex with men (MSM) were recruited from Beijing city. Subjects were divided into two groups according to the length of HIV-1 infection: 1–3 years (n = 22) and >3 years (n = 13) (Table 2). All MSM subjects were naïve of antiretroviral therapy.

**Table 2:** Characteristics of HIV-1-infected MSM recruited in the study

Characteristics	Duration of HIV-1 infection	
	1–3 years	>3 years
Number	22	13
Age (years)^†^	30.2 (6.6)	34.5 (7.0)
Deduced infection time (years)^†^	2.0 (0.6)	5.6 (2.1)
HIV RNA (copies/ml)^†^	25,611 (30,255)	27,657 (30,144)
CD4 cells/μl^†^	356 (105)	348 (241)
CD8 cells/μl^†^	924 (312)	874 (247)

^†^ Mean ± standard deviation (SD)

### CD16/CD32 surface staining

To compare the surface expression of CD16 and CD32 on circulating CD56+ T cells, CD56+ NK cells and CD56− T cells, PBMCs from 10 healthy controls were stained with anti-CD3 Pacific Blue (clone UCHT1), anti-CD56 PE-Cy7 (clone B159), anti-CD16 APC-Cy7 (clone 3G8), and anti-CD32 PerCP-eFluor 710 (clone 6C4). All antibodies were purchased from BD Biosciences (San Diego, CA, USA) except CD32 from eBiosciences (San Diego, CA, USA). Samples were analyzed on BD FACS Fortessa (BD Biosciences, San Jose, CA, USA) and the frequencies of CD16+ or CD32+ cells in CD56+ T cells, CD56+ NK cells and CD56− T cells were calculated and compared.

### Image analysis of CD16/CD32 expression

To identify the expression of CD16 and CD32 on CD56+ T cells, CD56+ NK cells and CD56− T cells at single-cell level, 1 × 10^5^ PBMC from three healthy controls were stained with anti-CD3 Pacific Blue (clone UCHT1), anti-CD56 PE-Cy7 (clone B159), anti-CD16 APC-Cy7 (clone 3G8) and anti-CD32 PerCP-eFluor 710 (clone 6C4) and were run on an ImageStream^χ^ MarkII system (Amnis Corporation, Seattle, WA, USA). 20,000 events were collected for each sample and single color control was used to create a compensation matrix to correct for spectral overlap. Collected data were analyzed using IDEAS 3.0 software (Amnis Corporation, Seattle, WA, USA).

### CD56+ T cells sorting

PBMCs were stained with anti-CD3 Pacific Blue (clone UCHT1, BD Biosciences), anti-CD56 PE-Cy7 (clone B159, BD Biosciences) for 30 min at room temperature. CD56+ T cells, CD56+ NK cells and CD56− T cells were sorted by BD FACS AriaIII (BD Biosciences, San Jose, CA) and only cells with purity >95% were used in subsequent experiments.

### Activation of purified CD56+ T cells by CD16/CD32 cross-linking

1 × 10^5^ purified CD56+ T cells from healthy controls were stimulated with 10 μg/ml of purified anti-CD16 antibody (clone 3G8, Santz Cruz biotechnology, Santa Cruz, CA, USA) or 10 μg/ml purified anti-CD32 antibody (clone 3D3, Santz Cruz biotechnology) or mouse IgG1(κ) (clone X40, BD Biosciences) isotype control for 30 min on ice. Cells were washed to remove unbound antibody and incubated with 10 μg/ml of goat anti-mouse IgG1 F(ab′)_2_ for 5 h (Santz Cruz biotechnology, Santa Cruz, USA) at 37 °C. Cells were washed and stained with anti-CD3 Pacific Blue (clone UCHT1), anti-CD56 PE-Cy7 (clone B159) and anti-CD107a PE-Cy5 (clone H4A3) and fixed by 2% paraformaldehyde (PFA). All data were acquired on BD FACS Fortessa (BD Biosciences, San Jose, CA, USA) and analyzed by FlowJo software (Treestar, Ashland, OR, USA).

### Evaluation of CD56+ T-mediated nonspecific ADCC activity

The nonspecific ADCC assay was performed as previously described, in which the mouse mastocytoma cell line P815 was used as target cells [27]. Briefly, PBMC were stimulated with P815 cells alone or P815 cells/P815-specific Abs (P815/Abs) complex (1:100 dilution of polyclonal rabbit anti-mouse lymphocyte serum, Accurate Chemical & Scientific Corp., Westbury, NY) at an E:T ratio of 10:1. Brefeldin-A (10 μg/ml, Sigma, St Louis, MO, USA), GolgiStop (5 μg/ml, BD Biosciences) and anti-CD107a PE-Cy5 (clone H4A3, BD Biosciences) were added immediately to cell medium and incubated for 6 h. Cells were fixed by 2% PFA and stained with anti-CD3 Pacific Blue (clone UCHT1), anti-CD56 PE-Cy7 (clone B159), anti-CD16 APC-Cy7 (clone 3G8), anti-CD4 PE (clone RPA-T4), anti-CD8 APC (clone RPA-T8), and anti-IFNγ FITC (clone 25,723.11). To evaluate the response time of antibody-dependent response mediated by CD56+ T and NK cells, PBMC were cocultured with P815/Abs for 2, 4 and 6 h and fixed and stained as above. All data were acquired on BD FACS Fortessa (BD Biosciences, San Jose, CA, USA) and analyzed by FlowJo software (Treestar, Ashland, OR, USA).

### Rapid fluorometric assay to test cytotoxicity mediated by CD56+ T cells

Based on methods adapted from a rapid fluorometric ADCC (RFADCC) assay [44], 1 × 10^6^ target cells (P815) were double-stained with 5 μM PKH-26 (Sigma, St. Louis, MO, USA) and 5 μM 5-(and-6)-carboxyfluorescein diacetate succinimidyl ester (CFSE) (Molecular Probes, Eugene, OR, USA). Purified CD56+ T cells, NK cells and T cells were used as effector cells and incubated with P815/Abs complex or P815 alone in a microtiter plate with an E:T ratio of 1:1. After 10 h incubation, cells were washed and fixed in cold 2% paraformaldehyde. Samples were run on BD FACS Fortessa (BD Biosciences, San Jose, CA, USA) and data were analyzed by FlowJo software (Treestar, Ashland, OR, USA).

### HIV-1 specific peptides

HIV-1 gag, pol, env solid-phase peptide pools containing 20-mers (overlapping by 10 amino acids) from HIV-1 strain CN54 were synthesized to 95% purity by Bio-Scientific Co., Shanghai, China. Peptides were dissolved to a concentration of 1 mg/ml in RPMI 1640 containing 10% dimethyl sulphoxide (DMSO) as stock solutions, and working solutions were obtained by further dilution to a final concentration of 1 μg/ml.

### CD56+ T-mediated HIV-1-specific ADCC assay

Whole blood samples were incubated with HIV-1 gag, pol and env peptide pools respectively at 37 °C for 5 h. Brefeldin-A (10 μg/ml, Sigma), GolgiStop (5 μg/ml, BD Biosciences) and anti-CD107a APC (clone H4A3, 20 μl/ml, BD Biosciences) were added immediately to the cell medium and incubated for 6 h. Samples incubated with phorbol-12-myristate-13-acetate (PMA) (1 mg/ml) plus ionomycin (1 mg/ml) were used as positive controls and samples incubated with DMSO (1 mg/ml, Sigma) were used as negative controls. Samples were surface-stained with live/dead dye Amcyan (Invitrogen, Carlsbad, CA, USA), anti-CD3-PerCP (clone SK7) and anti-CD56-PE (clone B159), and then lysed, permeabilized and intracellularly stained with anti-IFNγ Alexa Fluor700 (clone B27, BD Biosciences) and anti-CD107a APC (clone H4A3). All data were acquired on BD FACS Fortessa (BD Biosciences, San Jose, CA, USA) and analyzed by FlowJo software (Treestar, Ashland, OR, USA).

### ELISA assay

1 × 10^5^ sorted CD56+ T cells were co-cultured with 1 × 10^5^ P815 cells alone, 1 × 10^5^ P815 cells plus P815-specific Abs, or medium alone for 24 h at 37 °C. The level of secreted IFNγ in culture supernatants was detected by ELISA (cat# 88-7316, eBioscience, San Diego, CA, USA) according to the manufacturer’s instruction. The sensitivity of the ELISA kit was 4 pg/ml.

### Identification of peripheral iNKT cells

Human peripheral invariant NKT (iNKT) cells were characterized as CD3+ T cells expressing a specific TCR (Vα24-Jα18-Vβ11). To identify iNKT cells, PBMCs were surface-stained with anti-CD3 Pacific Blue (clone UCHT1, BD Biosciences) and anti-Vα24-Jα18 FITC (clone 6B11, BD Biosciences). CD3+ Vα24-Jα18+ double positive cells were defined as iNKT cells. To detect CD16, CD161 and CD69 expression on iNKT and CD56+ T cells, PBMC were surface-stained with anti-CD3 Pacific Blue, anti-Vα24-Jα18 FITC (clone 6B11), anti-CD56 PE-Cy7 (clone B159), anti-CD16 (clone eBioCB16, eBioscience), anti-CD161 (clone DX12, BD Biosciences) and anti-CD69 (clone FN50, BD Biosciences). Samples were analyzed on BD FACS Fortessa (BD Biosciences, San Jose, CA, USA) and the frequencies of CD16+ or CD32+ cells in CD56+ T cells, CD56+ NK cells and CD56− T cells were calculated and compared.

### Recovery of impaired antibody-dependent CD56+ T cell responses in HIV+ subjects by MMP inhibitor

1 × 10^5^ purified DN CD56+ T cells were cultured in interleukin-2-supplemented medium in the presence or absence of broad-spectrum MMP inhibitor GM6001 (5 mM) or DMSO for 0, 3, 6 and 12 h. CD16 expression (MFI) was detected by flow cytometry at each point. Additionally, changes in P815/Ab-stimulated purified CD56+ T cell responses (at an E:T ratio of 1:1) following GM 6001 (50 μM) (Sigma) treatment was analyzed after 6 h of coculture. An equivalent volume of DMSO (Sigma) was used as control. Cells were washed/stained as described above and fixed in cold 2% PFA. All data were acquired on BD FACS Fortessa (BD Biosciences, San Jose, CA, USA) and analyzed by FlowJo software (Treestar, Ashland, OR, USA).

To determine whether GM6001 could restore the capacity of dysfunctional CD56+ T cell-medicated ADCC in HIV-1 infected subjects, purified DN (CD4-CD8-) CD56+ T cells were cultured in interleukin-2 (IL2)-supplemented medium in the presence of 5 μM GM6001(Santz Cruz biotechnology, Santa Cruz, CA, USA) or an equivalent amount of DMSO vehicle alone. The degranulating capacities of CD56+ T cells following stimulation with P815 cells or Ab-coated P815 cells were analyzed and compared. After stained with anti-CD107a PE-Cy5 (clone H4A3, BD Biosciences) and anti-IFNγ FITC (clone 25,723.11, BD Biosciences), cells were washed by PBS and fixed by 2% paraformaldehyde (PFA). All data were acquired on BD FACS Fortessa and analyzed by FlowJo software.

### Statistical analysis

All the statistical and graphic analyses were performed using GraphPad Prism 5.0 (GraphPad Software, La Jolla, CA, USA) or Microsoft Excel 2007. Comparisons between groups were performed using Mann–Whitney *U* test, nonparametric *t* test, or Wilcoxon matched-pairs signed rank test when necessary. The Spearman’s correlation test was used to evaluate correlations between groups. All *P* values were two-tailed and considered significant when lower than 0.05.

### Additional files

**Additional file 1: Figure S1.** Purity detection of sorted CD56+ T cells. CD56+ T cells were sorted by BD FACSAriaIII and detected for purity with BD FACS Fortessa. The figure showed a representative result for purity test (96.4%).

**Additional file 2: Figure S2.** Comparison of the response time of non-specific ADCC mediated by CD56+ T and CD56+ NK cells. **a** Levels of CD107a expression and IFNγ production were detected from CD56+ T cells and CD56+ NK cells incubated with P815 plus Abs for 2, 4, 6 h by flow cytometry. **b** Comparison of the frequencies of CD107a+ cells and IFNγ+ cells between CD56+ T cells and CD56+ NK cells in different responding time (n = 10).

**Additional file 3: Figure S3.** Comparison of the TCR-dependent responses mediated by CD56+ T and CD56− T cells. **a** PBMC cells from HIV-1-infected patients were activated by env, gag and pol peptides for 6 h. SEB activation and medium alone were set as positive and negative controls respectively. Intracellular IFNγ production was detected from activated CD56+ T cells and CD56− T cells by flow cytometry. **b** Comparison of the frequencies of IFNγ+ cells between activated CD56+ T cells and CD56− T cells from HIV-1-infected patients (n = 10).

## Abbreviations

ADCC: antibody-dependent cellular cytotoxicity
NK: natural killer
HIV-1: human immunodeficiency virus type-1
HCV: hepatitis C virus
MMP: matrix metalloprotease
AIDS: acquired immune deficiency syndrome
iNKT: invariant NKT
TCR: T cell receptor
α-GC: α-galactosylceramide
MHC: major histocompatibility complex
PBMC: peripheral blood mononuclear cell
IFNγ: interferon-γ
FPDs: former plasma donors
MSM: men who have sex with men
DN: double negative subset
HESN: HIV-exposed seronegative
6B11+: Vα24-Jα18+
NKR: nature killer cell receptor
CAP/CTM: Cobas Ampliprep/CobasTaqman
HBsAg: hepatitis B surface antigen
PFA: paraformaldehyde
DMSO: dimethyl sulphoxide
PMA: phorbol-12-myristate-13-acetate
