# Supplementary figures and images for: Antibody-dependent CD56+ T cell responses are functionally impaired in long-term HIV-1 infection
Source: Retrovirology. 2016 Nov 4;13:76. doi: 10.1186/s12977-016-0313-6 (PMC5097383; doi:10.1186/s12977-016-0313-6)

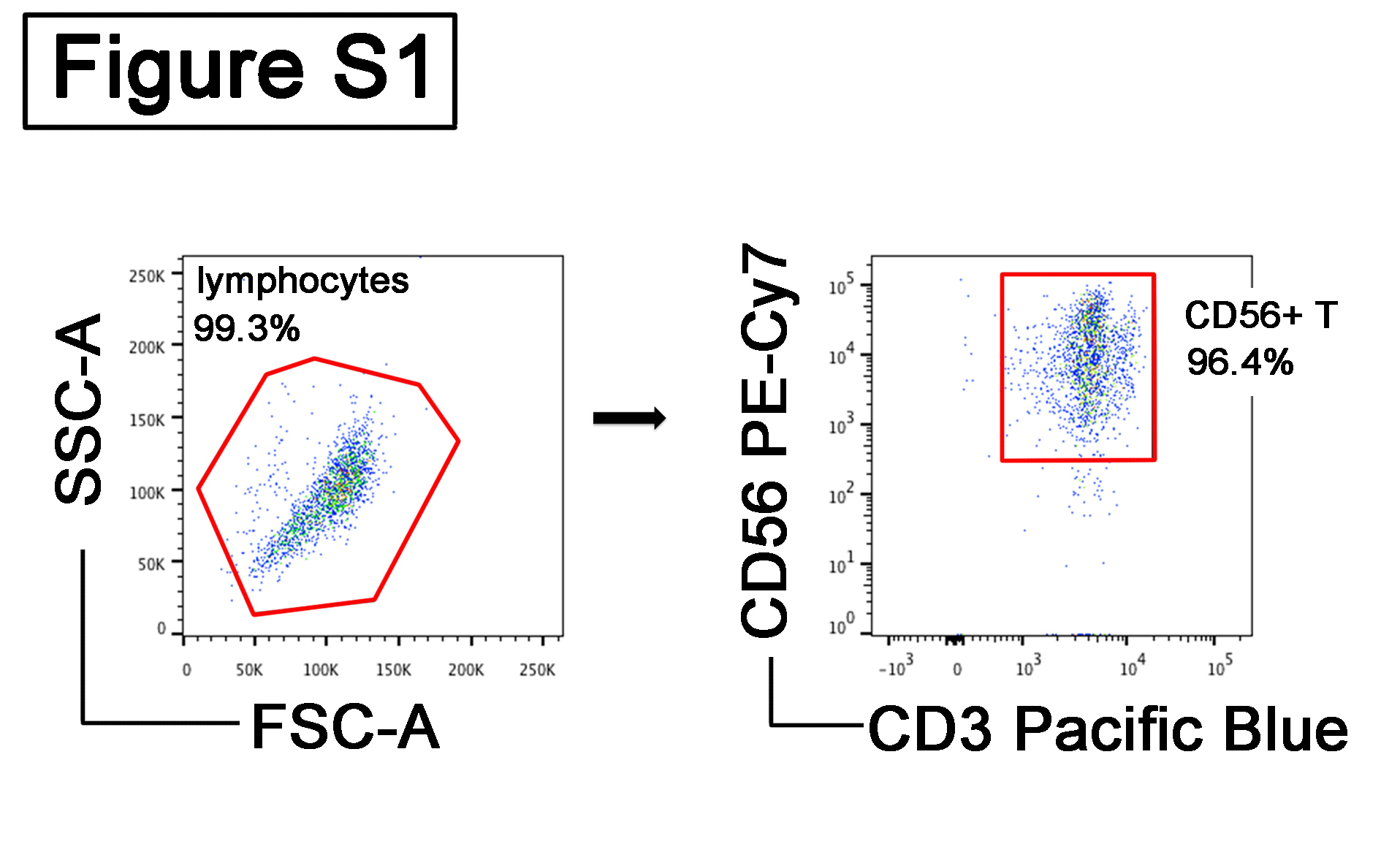

Supplement: Supplementary file 1 — Additional file 1: Figure S1. Purity detection of sorted CD56+ T cells. CD56+ T cells were sorted by BD FACSAriaIII and detected for purity with BD FACS Fortessa. The figure showed a representative result for purity test (96.4%). [file 12977_2016_313_MOESM1_ESM.jpg]

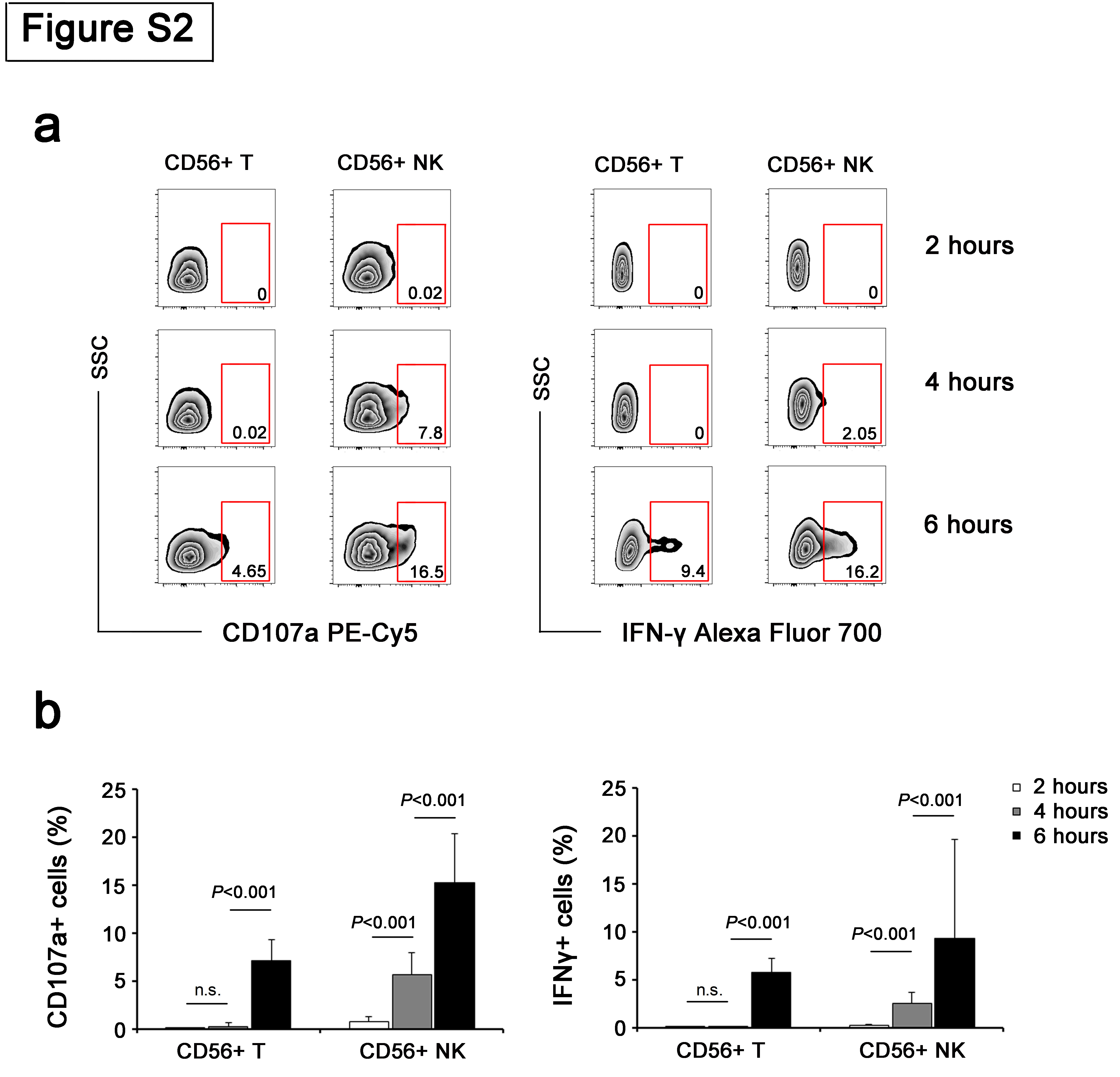

Supplement: Supplementary file 2 — Additional file 2: Figure S2. Comparison of the response time of non-specific ADCC mediated by CD56+ T and CD56+ NK cells. a Levels of CD107a expression and IFNγ production were detected from CD56+ T cells and CD56+ NK cells incubated with P815 plus Abs for 2, 4, 6 h by flow cytometry. b Comparison of the frequencies of CD107a+ cells and IFNγ+ cells between CD56+ T cells and CD56+ NK cells in different responding time (n = 10). [file 12977_2016_313_MOESM2_ESM.jpg]

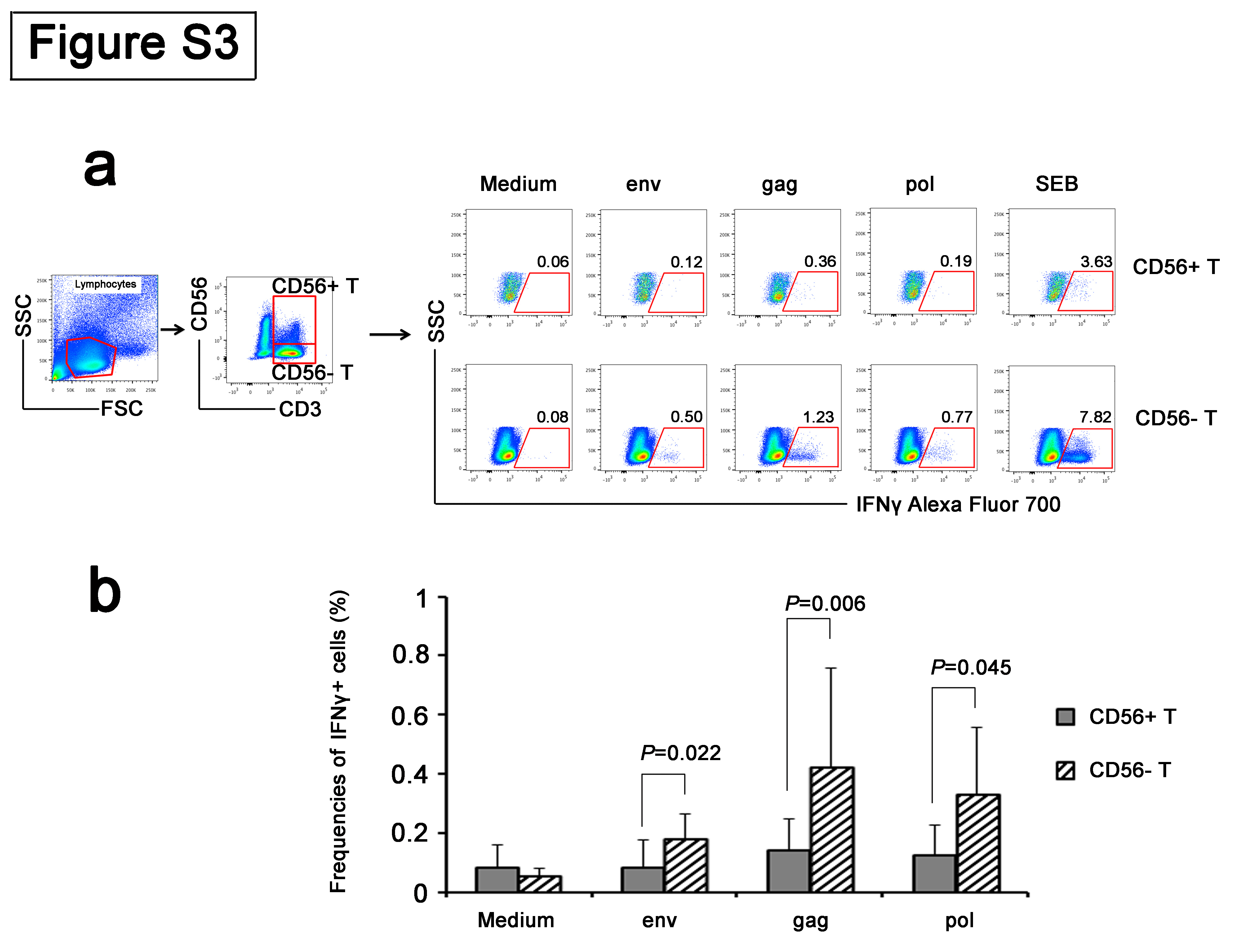

Supplement: Supplementary file 3 — Additional file 3: Figure S3. Comparison of the TCR-dependent responses mediated by CD56+ T and CD56− T cells. a PBMC cells from HIV-1-infected patients were activated by env, gag and pol peptides for 6 h. SEB activation and medium alone were set as positive and negative controls respectively. Intracellular IFNγ production was detected from activated CD56+ T cells and CD56− T cells by flow cytometry. b Comparison of the frequencies of IFNγ+ cells between activated CD56+ T cells and CD56− T cells from HIV-1-infected patients (n = 10). [file 12977_2016_313_MOESM3_ESM.jpg]
